# Multi level effects of Sanghuang Tongxie Formula on type 2 diabetes rats: a comprehensive analysis from intestinal bacteria to metabolome and transcriptome

**DOI:** 10.3389/fendo.2025.1562105

**Published:** 2025-11-11

**Authors:** Jiaxuan Liu, Sufen Bai, Chenxi Wu, Chunyu Tian, Qianru Fu, Xiujuan Gao, Biwei Zhang, Ji’an Li, Xiumei Cheng, Xiaojin La

**Affiliations:** 1 College of Traditional Chinese Medicine, North China University of Science and Technology, Tangshan, China; 2 College of Traditional Chinese Medicine, Hebei University of Chinese Medicine, Shijiazhuang, Hebei, China

**Keywords:** Sanghuang Tongxie Formula, 16S, metabolome, RNA-seq, type 2 diabetes mellitus

## Abstract

**Objective:**

Type 2 diabetes mellitus (T2DM) is a chronic metabolic disorder characterized by progressive β-cell dysfunction and peripheral insulin resistance, leading to dysregulated glucose homeostasis and sustained low-grade inflammation. This study aimed to evaluate the therapeutic efficacy of the traditional Chinese medicine, Sanghuang Tongxie Formula (SHTX), in a rat model of T2DM and to elucidate its underlying mechanisms by analyzing gut microbiota composition, metabolic pathways, and gene expression profiles using metabolomics and transcriptomics.

**Methods:**

A Type 2 diabetes mellitus model was established in Sprague-Dawley (SD) rats through a high-fat diet combined with intraperitoneal injection of streptozotocin (STZ). The therapeutic effects of SHTX on the gut microbiota, metabolic pathways, and gene expression were evaluated through high-throughput sequencing and comprehensive metabolomics analysis.

**Results:**

SHTX treatment significantly altered the gut microbiota composition in T2DM rats, increasing the relative abundance of Bacteroidetes while decreasing that of Firmicutes, which was closely correlated with improved insulin sensitivity. Metabolomics analysis revealed that SHTX modulated the glycerophospholipid metabolic pathway, resulting in significant alterations in key metabolites. Notably, genes involved in glycerophospholipid metabolism, such as Plin2 and PLD1, showed marked changes in expression in response to SHTX treatment. Additionally, transcriptomic profiling demonstrated that SHTX profoundly impacted multiple metabolic and regulatory pathways in the liver, with a particular focus on those associated with glycerophospholipid metabolism.

**Conclusion:**

These results suggest that SHTX treatment may improve metabolic homeostasis in T2DM by modulating gut microbiota and several key metabolic pathways, particularly glycerophospholipid metabolism. However, the study has several limitations, including the reliance on animal models and the incomplete elucidation of the underlying mechanisms. Further studies are needed to confirm these findings and explore the molecular mechanisms of SHTX therapy. Additionally, clinical trials are necessary to assess the therapeutic potential and efficacy of SHTX in human T2DM patients.

## 1 Introduction

Diabetes mellitus (DM) has emerged as a major global public health concern. According to the 2021 report from the International Diabetes Federation (IDF), the global population of individuals living with diabetes is approaching 550 million, with projections indicating further increases by 2030 (1). Diabetes significantly compromises patients’ quality of life, increases the risk of complications such as cardiovascular disease, renal disease, retinopathy, and neuropathy, and imposes a considerable financial burden on healthcare systems worldwide. Type 2 diabetes mellitus (T2DM) accounts for over 90% of all diabetes cases, primarily characterized by insulin resistance, which leads to disturbances in glucose and lipid metabolism and results in persistent hyperglycemia (2). Recent advances in understanding the pathophysiology of diabetes have highlighted the limitations of conventional treatments, particularly the development of during long-term pharmacological management of diabetes, some patients may develop drug tolerance, leading to diminished glycaemic control efficacy. Furthermore, different hypoglycaemic agents may induce adverse reactions such as hypoglycaemia, weight fluctuations, and gastrointestinal discomfort (3). As a result, there has been an increasing focus on developing more effective treatment strategies with fewer side effects.

Traditional Chinese Medicine (TCM), with its extensive therapeutic history and rich pharmacological resources, has gained recognition as an alternative therapeutic approach for the management of diabetes. The Sanghuang Tongxie Formula, a traditional TCM formulation, is composed of Huangqi (*Radix Astragali seu Hedysari)*, Sangbaipi (Cortex Mori), Huanglian (Coptis Chinensis), Shanzhuyu (Cornus officinalis), Houpo (Magnolia officinalis),et al. The diverse active constituents within these herbs may exert therapeutic effects by modulating various physiological pathways within the body. In recent years, gut microbiota, metabolomics and transcriptomics have garnered significant attention as pivotal domains for exploring disease mechanisms and drug actions. Numerous studies have preliminarily revealed their close association with type 2 diabetes. Astragalus polysaccharides have been reported to improve GLP-1 secretion and intestinal barrier function by enriching beneficial bacteria such as Akkermansia and Faecalibaculum and increasing short-chain fatty acid production (4). Previous studies have shown that Sanghuang Tongxie Formula alleviates insulin resistance in type 2 diabetes by shifting nutrient metabolism toward lipid anabolism while activating the PI3K/Akt signaling pathway (5). Moreover, berberine, the representative active component of Coptis chinensis in the formula, can modulate multiple signaling pathways by acting upon core inflammation-related genes such as IL-6, VEGF-A and TNF. This thereby ameliorates the chronic inflammatory state associated with type 2 diabetes and protects pancreatic islet function (6).

Although preliminary studies have explored the effects of medicinal components in the SHTX on regulating glucose and lipid metabolism, improving insulin resistance, and exerting anti-inflammatory actions, its overall mechanism of action remains unclear. This gap primarily stems from the complexity of traditional Chinese medicinal formulas, which contain multiple active constituents whose effects involve intricate regulation across multiple targets and signaling pathways. Traditional single-mechanism studies often focus on localized effects, failing to elucidate the synergistic interaction patterns and key molecular events of compound formulations within the biological organism.

To address this gap, multi-omics approaches such as gut microbiome analysis, non-targeted metabolomics and transcriptomics provide comprehensive tools to elucidate the integrated mechanisms of action within complex Chinese herbal formulas. Multi-omics enables synchronous collection of cross-level data within a single research framework. By integrating information from different biological levels, it systematically reveals the synergistic effects of drug interventions on microbial community structure, metabolic network remodeling, and host gene expression regulation (7). Particularly for systemic metabolic disorders such as diabetes, the interconnected network between gut microbiota, metabolic products, and host genes plays a pivotal role in disease progression and therapeutic response. Multi-omics integration effectively captures this intricate dynamic process.

Therefore, this study employs multi-omics integrated analysis as its core strategy to systematically evaluate the intervention effects of SHTX and its potential molecular mechanisms in a rat model of type 2 diabetes mellitus. Based on prior research and our group’s preliminary work, we hypothesize that SHTX may promote the maintenance of glucose and lipid metabolic homeostasis by optimizing gut microbiota composition and improving the functional status of gut-liver metabolic signaling pathways. The primary objective of this study is to validate this hypothesis and construct a multi-level molecular mechanism network elucidating how SHTX exerts its effects in the T2DM state, thereby providing scientific rationale for its clinical application.

## 2 Materials and methods

### 2.1 Preparation and quality assurance of SHTX extracts

All herbs used in this study (**Table 1**) were sourced from Beijing Tongrentang Tangshan Pharmacy Co., Ltd. The specific herbs included Sangbaipi (batch no. 20191120), Huanglian (batch no. 26819202), Houpao (batch no. 190901), Zhimu (batch no. 20200621), Yuzhu (batch no. 20200605), Cornus officinalis (batch no. 20200801), Lycoris radiata (batch no. 20200202), Astragalus membranaceus (batch no. 20180613), Pueraria lobata (batch no. 190901), and Atractylodes macrocephala (batch no.2020026). The identity and quality of all herbs were verified by Prof. Chunyu Tian from the School of Traditional Chinese Medicine at North China University of Science and Technology prior to their use, ensuring their authenticity, quality, and therapeutic efficacy.

**Table 1:** Composition of Sanghuangtongxie decoction (SHTX) and the characteristics of each constituent drugs.

Drug name	English name	Medicinal part	Weight (g) in BHID
Sang BaiPi	Mori Cortex	Bark	15
Huang Lian	*Coptidis Rhizoma*	Rhizome	15
Hou Po	*Mag.Officnalis Cortex*	Bark	10
Zhi Mu	Anemarrhenae rhizoma	Rhizome	5
Yu Zhu	Pol.Odorati Rhizoma	Rhizoma	5
Shan Zhuyu	*Cornifructus*	Fruit	5
Lai Fuzi	*Raphani Semen*	Seed	5
Huang Qi	*Astragali Radix*	Root	5
Ge Gen	*Pue.Lobatae Radix*	Root	5
Cang Zhu	*Atractylodes chinensis*	Root	5

To prepare the SHTX extract, 75 g of the combined herbs were processed according to the following procedure: (1) The herbs were soaked in distilled water for 2 hours; (2) The mixture was gently boiled and then simmered at low heat for 1 hour to extract the herbal broth; (3) A sufficient volume of distilled water was added to the residue, and the boiling process was repeated. The broths from both extractions were then combined. The resulting mixture was transferred to a flat plate and placed in a water bath maintained at 90°C or higher, allowing the broth to evaporate until the desired concentration was achieved. The concentrated SHTX extract was stored at 4°C. Before use, the extract was reconstituted with distilled water to the appropriate concentration for application. The preparation and quality control procedures for the Sanghuang Tongxie Formula (SHTX) extract are detailed in **Supplementary Figure 1**.

### 2.2 LC-MS/MS profiling technique

LC-MS/MS analysis was performed using a UPLC-Orbitrap-HRMS platform (Thermo Fisher Scientific) coupled with a Waters ACQUITY UPLC HSS T3 column (100 mm × 2.1 mm, 1.8 μm). The mobile phase consisted of acetonitrile (A) and an aqueous solution containing 0.1% formic acid (B). Separation was achieved using a gradient elution program as follows: from 0 to 1 min, A was set to 0%; from 1 to 2 min, A was gradually increased to 20%; from 2 to 12 min, A was held at 50%; from 12 to 15 min, A increased from 50% to 95%; and from 15 to 20 min, A was increased from 95% to 100%. The flow rate was set at 0.4 mL/min, and the column temperature was kept at 30°C. A sample injection volume of 5 μL was used for each analysis. The electrospray ionization (ESI) source alternated between positive and negative ion modes, with a scanning mass range of 100–1000 m/z. For quality control, pooled QC samples were prepared by mixing equal aliquots from each SHTX extract and analyzed every 8 injections to monitor retention time and signal stability. L-2-chlorophenylalanine (2 μg/mL, ≥ 98%, Sigma-Aldrich) was added to all samples before extraction as an internal standard for normalization. Method validation included assessment of mass accuracy (Δm/z < 5 ppm) and peak area precision (RSD < 15% in QC replicates), all meeting acceptance criteria. Data acquisition and analysis were performed using Thermo Xcalibur software (version 4.0).

### 2.3 Animal experiments

Thirty healthy male Sprague-Dawley (SD) rats, aged 4 weeks and weighing 260 ± 20 g, were used in this study. All animals were sourced from HuaFuKang Biotechnology Co. The rats were housed under specific pathogen-free (SPF) conditions at the Laboratory Animal Center of North China University of Science and Technology, China. The environmental conditions were strictly controlled, with a 12-hour light/dark cycle, a room temperature of 25°C, and relative humidity maintained between 50% and 60%. To minimize potential confounding effects from circadian rhythms, all experiments were conducted at the same time each morning. All animal experiments conducted in this research institute strictly adhere to the ARRIVE 2.0 guidelines for reporting animal research and received approval from the Animal Ethics Committee of North China University of Science and Technology (2022028).

### 2.4 Chemicals and equipment

#### 2.4.1 Principal reagents

Modeling and intervention-related reagents: Streptozotocin (STZ, purity ≥ 98%, Sigma-Aldrich, USA, Catalog No. S0130) for inducing type 2 diabetes mellitus (T2DM) models; its buffer was prepared using citric acid (analytical grade, Beijing Boaigang Biotechnology Co., Ltd., Catalog No. 100112) and sodium citrate (analytical grade, Beijing Boaigang Biotechnology Co., Ltd., Catalog No. 1001113) (pH 4.5); High-fat diet (containing 60% fat, 20% protein, 20% carbohydrates, Beijing Boaigang Biotechnology Co., Ltd., Cat. No. 12492M) was administered for pre-modeling feeding; Protein detection reagents: Primary antibodies for Western blot analysis included β-actin monoclonal antibody (dilution 1:1000, Biyun Tian Biotechnology Co., Ltd, Catalog number AF0003), and polyclonal antibodies against Plin2 (1:1000, Abcam, catalog number ab108323) and PLD1 (1:1000; CUSABIO; CSB-YP018144RA)., and secondary antibody: horseradish peroxidase (HRP)-labeled goat anti-rabbit antibody (dilution 1:5000, Biyun Tian Biotechnology Co., Ltd, Catalog number A0208). These were collectively employed for the specific detection of target proteins.

#### 2.4.2 Principal instruments

A blood glucose meter (Sannuo, 1QJBOR17850) was employed to measure fasting blood glucose levels in rats; a tissue dehydrator (LEICA, Germany, TP1020) and a panoramic tissue cell quantitative analysis system (ZEISS, Germany, TissueGnostics) were utilized for pathological observation of liver tissue sections and lipid droplet counting; Real-time fluorescent quantitative polymerase chain reaction (qRT-PCR) instrument (BIO RAD, T100TM Thermal Cycler) for detecting mRNA expression levels of Plin2 and PLD1 genes in liver tissue; Electrophoresis power supply (Thermo Fisher Scientific, USA, PS2108); Electrotransfer apparatus (Thermo Fisher Scientific, USA, Mini Blot Module 072021); Chemiluminescent imaging system (BIO-RAD, USA, ChemiDoc™ CXRS+System) for detecting Plin2 and PLD1 protein expression levels in liver tissue.

### 2.5 HFD/STZ-induced type 2 diabetes mellitus rat model

Animals were randomly assigned to two groups using a random number table: a control group (n = 10) and a T2DM group (n = 20). After 4 weeks of high-fat diet (HFD) feeding, type 2 diabetes mellitus (T2DM) was induced in the T2DM group by a single intraperitoneal injection of streptozotocin (STZ, Sigma-Aldrich, USA) at a dose of 35 mg/kg body weight. The STZ was dissolved in a 0.1 M citric acid/sodium citrate buffer and adjusted to pH 4.5. The success of diabetes induction was confirmed by measuring fasting blood glucose levels two days post-injection. Rats exhibiting blood glucose levels above 16.7 mmol/L for three consecutive days were considered diabetic and were included in the subsequent experiments (8). The entire experiment was conducted using a single-blind design.

Following the establishment of the T2DM model, rats were randomly divided into two subgroups: the model group (n = 10) and the SHTX group (n = 10). Rats in the SHTX group were administered Sanghuang Tongxie Formula (SHTX) extract via oral gavage at a dose of 14 g/kg body weight, which was pharmacologically determined to be seven times the standard human dosage.

### 2.6 Measurement of physiological characteristics

Physiological parameters, including body weight, food and water intake, and fasting blood glucose levels, were measured weekly for 8 weeks. Blood samples were collected from the abdominal aorta for further analysis.

### 2.7 Histology of the liver and intestine

Liver and intestine tissue samples were collected immediately after euthanasia. The liver tissues were preserved in RNA preservation solution at 4°C overnight for subsequent RNA extraction and transcriptome analysis. Another portion of liver tissue was rapidly frozen in liquid nitrogen and stored at -80°C for future gene sequencing and Western blot analyses. Additionally, a section of liver and intestine tissue was fixed in 4% paraformaldehyde, embedded in paraffin, and cut into 4 μm-thick slices. The tissue sections were stained with hematoxylin-eosin (HE) and examined under a light microscope. Images were captured at 200× magnification and quantitatively analyzed using ImageJ software.

### 2.8 Examination of intestinal microbiota

Fecal samples were randomly selected from rats in each group and stored at -80°C for subsequent analysis. Genomic DNA was extracted from the fecal samples using SDS methods, and its purity and concentration were assessed using standard spectrophotometric techniques. According to the selected sequencing region, the V3–V4 hypervariable regions of the 16S rRNA gene were amplified using barcoded specific primers 341F (5′-CCTACGGGNGGCWGCAG-3′) and 806R (5′-GGACTACHVGGGTWTCTAAT-3′) and a high-fidelity DNA polymerase (Phusion^®^ High-Fidelity PCR Master Mix, New England Biolabs) (9). The PCR products were analyzed by 2% agarose gel electrophoresis, and the target bands were excised and purified using the AxyPrep DNA Gel Recovery Kit (Axygen, USA). Initial quantification was based on gel electrophoresis results, and the PCR products were further quantified using the QuantiFluor™-ST Blue Fluorescence Quantification System (Promega). Sequencing libraries were constructed using the NEBNext^®^ Ultra™ DNA Library Prep Kit for Illumina (NEB, USA). The quality of the libraries was evaluated using the Agilent Bioanalyzer 2100 and Qubit fluorometer. Paired-end sequencing (2 × 250 bp) was performed on the Illumina MiSeq platform according to the manufacturer’s instructions (10).

### 2.9 Methods and data analysis in gut microbiome research

Sequence data were quality-filtered, and taxonomic abundance tables were rarefied to an equal sequencing depth. Beta-diversity and alpha-diversity analyses were conducted, and group differences were assessed by permutational multivariate analysis of variance (PERMANOVA). Differential taxa were identified using the linear discriminant analysis effect size (LEfSe) algorithm, with an LDA score > 3.0 and *P* < 0.05 after false discovery rate (FDR) correction (Benjamini–Hochberg method).

### 2.10 Sequencing data processing and quality control

Raw paired-end reads were assigned to samples according to their unique barcodes. Adapter sequences and primers were removed, and overlapping paired-end reads were merged using FLASH. Quality filtering was performed to remove reads with ambiguous bases (N), short read length (<200 bp), or low average quality scores (Q-score <20). Chimeric sequences were detected and removed using UCHIME against the SILVA reference database. High-quality (“clean”) reads were retained for downstream analysis, with each sample achieving ≥50,000 clean reads and Good’s coverage >99%, ensuring sufficient sequencing depth and coverage.

### 2.11 Preparation of liver samples for metabolomics

Rat liver tissues were immediately frozen in liquid nitrogen following dissection. Approximately 80 mg of liver tissue was cut into small pieces and transferred into a 2 mL centrifuge tube containing 200 μL of water and five ceramic beads. The tissue was homogenized using a homogenizer to ensure complete disruption. Subsequently, add 800 μL of a methanol/acetonitrile mixture (2:2 v/v) to the homogenate for extraction. Centrifuge at 14,000g (4°C, 20 min) after precipitating at -20°C for 10 minutes. The supernatant was vacuum-dried, redissolved in acetonitrile/water (1:1, v/v), centrifuged again (14,000g, 4°C, 15 min), and analyzed by ultra-high-performance liquid chromatography-quadrupole time-of-flight mass spectrometry (UHPLC-Q-TOF MS). Raw data (.wiff.scan) were converted to mzXML format using ProteoWizard and subjected to peak detection in XCMS (centWave, m/z=25ppm, peak width=c(10,60), prefilter=c(10,100)), grouped (bw=5, mzwid=0.025, minfrac=0.5), and isotope/adduct annotated (CAMERA). Only features present in >50% of a sample group were retained. Metabolites were identified by retention time, mass accuracy (<25 ppm), MS/MS spectrum, and collision energy against an internal standard database, followed by manual validation. Identified results were reported with an MSI confidence level ≥ 2 (11).

### 2.12 Metabolomics research and data analysis

Instrumental signals were normalized to internal standards and total ion current, followed by log transformation and Pareto scaling. Multivariate analysis was performed using orthogonal partial least squares−discriminant analysis (OPLS−DA), with model validity assessed by permutation testing. Differential metabolites were defined by variable importance in projection (VIP) > 1.0 and FDR−adjusted *P* < 0.05.

### 2.13 Transcriptomics

Total RNA was extracted from the liver tissue of each rat. After assessing RNA quality and concentration, mRNA was enriched using Oligo(dT) magnetic beads. The mRNA was then fragmented randomly by adding the fragmentation buffer. First-strand cDNA was synthesized using six-base random primers (random hexamers), followed by second-strand cDNA synthesis with the addition of buffer, dNTPs, and DNA polymerase I. The double-stranded cDNA was purified using AMPure XP beads.

The purified cDNA was subjected to end repair, A-tail addition, and ligation of sequencing adapters. Fragment size selection was performed using AMPure XP beads. A final cDNA library was generated via PCR amplification. The library’s quality was assessed by measuring insert length and effective concentration. High-quality libraries were pooled based on the required sequencing depth and processed for high-throughput sequencing.

To evaluate the effect of SHTX on gene expression in the livers of T2DM rats, differentially expressed genes (DEGs) were identified with p-adjust < 0.05, |log_2_FC| > 1. Genes upregulated in the model group and downregulated in the SHTX group, or vice versa, were considered DEGs. DEGs were subjected to gene clustering, Gene Ontology (GO) analysis, and Kyoto Encyclopedia of Genes and Genomes (KEGG) enrichment analysis, accessible at KEGG.(accessible at http://www.genome.jp/kegg/).

### 2.14 Western blotting

Liver tissue from each rat was dissected and homogenized using a homogenizer with the addition of lysis buffer. The homogenized samples were then centrifuged at 12,000 rpm for 15 minutes at 4°C to separate the supernatant. The protein concentration in the supernatant was quantified using a BCA protein assay kit. The calculated protein extraction yield was 7.5 mg/g (n = 6 per group, with 3 technical replicates per sample). Protein samples were separated by 10% sodium dodecyl sulfate-polyacrylamide gel electrophoresis (SDS-PAGE) and transferred onto polyvinylidene difluoride (PVDF) membranes (0.22 µm pore size).

The membranes were blocked with 5% skimmed milk for 2 hours at room temperature and washed three times with Tris-buffered saline (TBST) containing Tween-20. The membranes were then incubated overnight at 4°C with primary antibodies against Plin2, PLD1 After three washes, the membranes were incubated with a secondary antibody at room temperature for 2 hours. Protein bands were detected using enhanced chemiluminescence (ECL) and quantified using ImageJ software. Each sample was measured in triplicate, and the average value was recorded.

### 2.15 Reverse transcription and quantitative polymerase chain reaction

Total RNA was extracted from rat liver tissue using the RNAeasy™ Animal RNA Extraction Kit (centrifugal column method) following the manufacturer’s instructions. RNA concentration and purity were assessed by spectrophotometry, with an expected A260/A280 ratio between 1.8 and 2.0.

cDNA synthesis was performed using a high-efficiency reverse transcription kit, such as SuperScript™ III (Thermo Fisher Scientific). The reaction mix included 1 μg of RNA, reverse transcriptase, dNTPs, Oligo(dT) primer, and reverse transcription buffer, with conditions optimized according to the kit specifications.

The synthesized cDNA was used as a template for quantitative polymerase chain reaction (qPCR) using a SYBR Green PCR kit (e.g., Applied Biosystems). Each qPCR reaction contained 10 μL of SYBR Green Master Mix, 0.5 μM primers, 1 μL of cDNA template, and nuclease-free water to a final volume of 20 μL. Primers were designed based on target genes, and the reaction conditions were as follows: an initial denaturation at 95°C for 3 minutes, followed by 40 cycles of 15 seconds denaturation at 95°C, 30 seconds annealing at 60°C, and 30 seconds extension at 72°C. Each sample was measured in triplicate to ensure reproducibility.

Relative gene expression was quantified using the ΔΔCt method, with GAPDH as an internal reference genes for normalization. Data analysis was performed using GraphPad Prism software.

### 2.16 Statistical analysis

Data are expressed as the mean ± standard deviation (SD). Prior to parametric testing, data were assessed for normality using the Shapiro–Wilk test and for homogeneity of variances using Levene’s test. For normally distributed data with equal variances, one-way analysis of variance (ANOVA) was performed to compare differences among groups, followed by Tukey’s *post hoc* test. If variances were not equal, Welch’s ANOVA with Games-Howell *post hoc* test was applied. All statistical tests were two-tailed, and *P* < 0.05 was considered statistically significant. Statistical analyses for conventional biochemical and physiological data were performed using SPSS 25.0 (IBM Corp., Armonk, NY, USA).

## 3 Results

### 3.1 LC-MS profiles of SHTX

**Figure 1:**
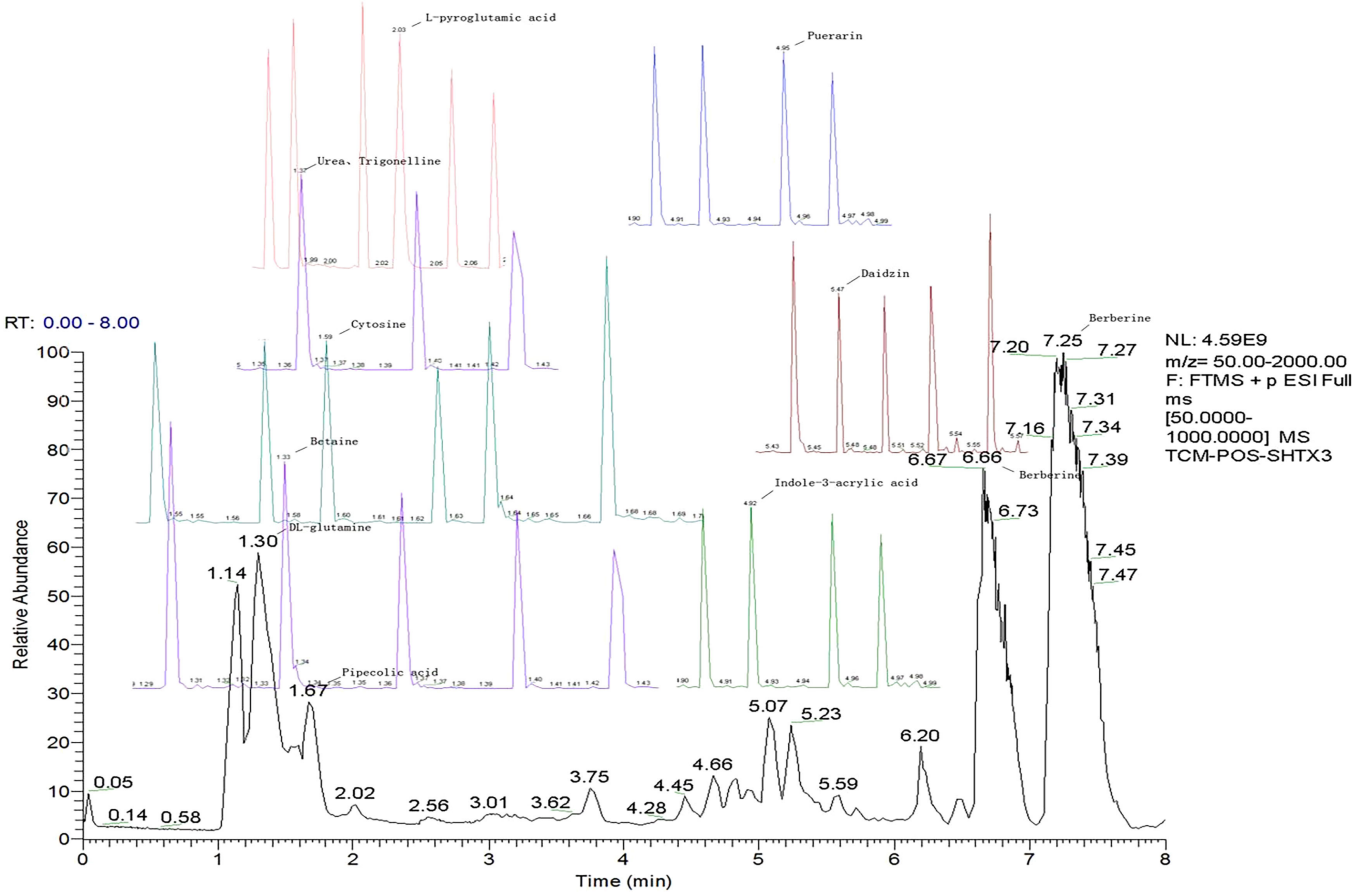
LC-MS chromatogram of SHTX sample spectral data is listed in **Table 2**. The x-axis shows retention time in minutes; the y-axis shows the intensity. black: Total ion chromatograms; red: L-pyroglutamic acid; purple: Urea, Trigonelline; green: Cytosine; violet: Betaine; blue:Puerarin; brown: Daidzin; sap green: Indole-3-acrylic acid.

LC-MS/MS analysis was conducted under optimized conditions, and the data, including peak retention times, UV range, and [MH] (m/z) values, were matched against a standard library. The total ion chromatograms revealed retention times (RT) for all identified compounds ranging from 1.14 to 7.47 minutes. The identified compounds, listed in **Table 2**, are shown in **Figure 1**.

**Table 2:** List of compounds detected in LC-MS.

S. No	Component name	Formula	Area (max.)	Observed RT (min)	Structural formula
1	Berberine	C_20_H_18_NO_4_^+^	4866557431	6.799	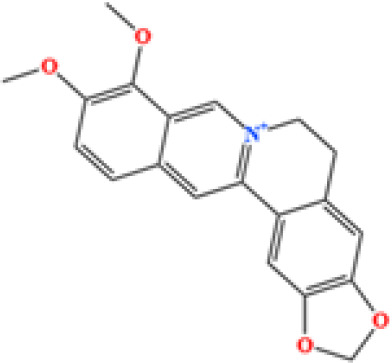
2	Ficine	C_20_H_19_NO_4_	10091661181	6.641	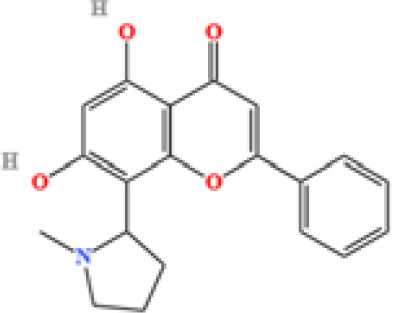
3	L-(+)-Arginine	C_6_H_14_N_4_O_2_	4675503282.07367	1.265	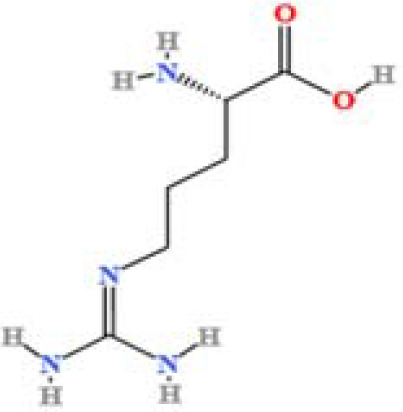
4	D-(+)-Proline	C_5_H_9_NO_2_	3903892004.07409	1.408	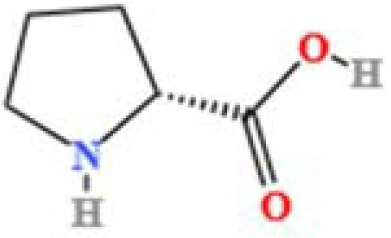
5	Choline	C_5_H_14_NO^+^	3121915280.48874	1.294	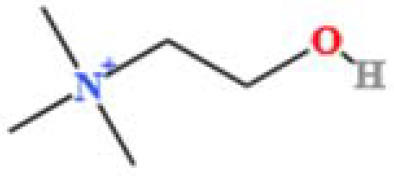
6	Puerarin	C_21_H_20_O_9_	1813722868.5104	4.957	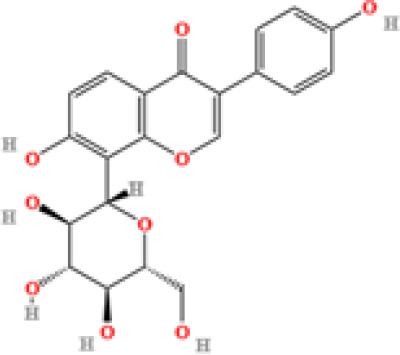
7	Betaine	C_5_H1_1_NO_2_	1164453658.40239	1.348	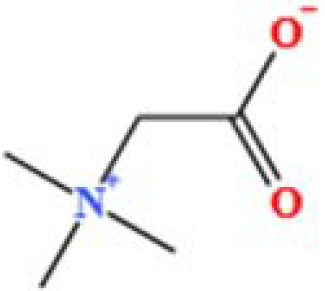
8	Terricollene B	C_17_H_22_O_7_	1100618298.41678	6.737	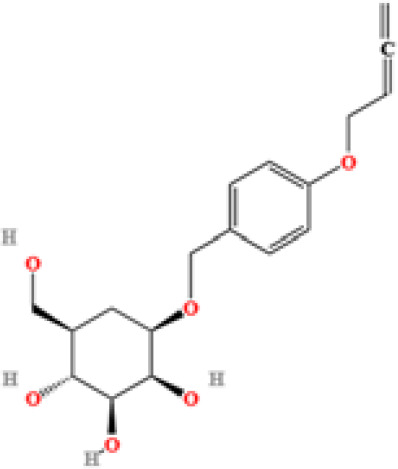
9	Indole-3-acrylic acid	C_11_H_9_NO_2_	973016241.8	4.484	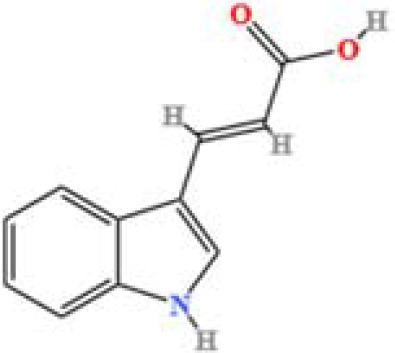
10	Neplanocin A	C_11_H_13_N_5_O_3_	764405755.615328	1.34	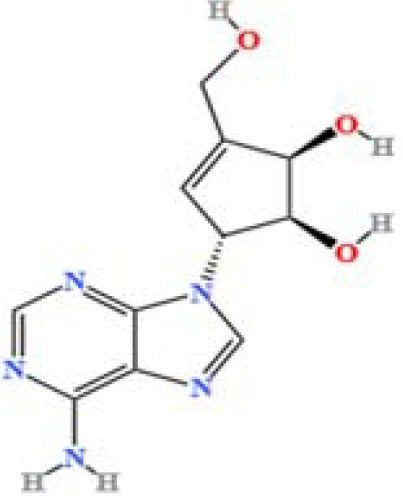
11	Pipecolic acid	C_6_H_11_NO_2_	695825938.1	1.687	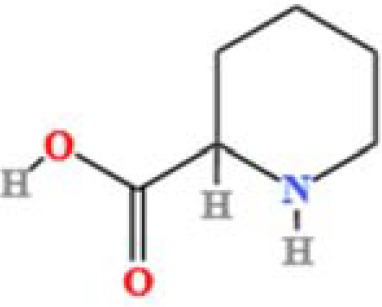
12	Aristeromycin	C1_1_H_15_N_5_O_3_	666365790.349053	1.35	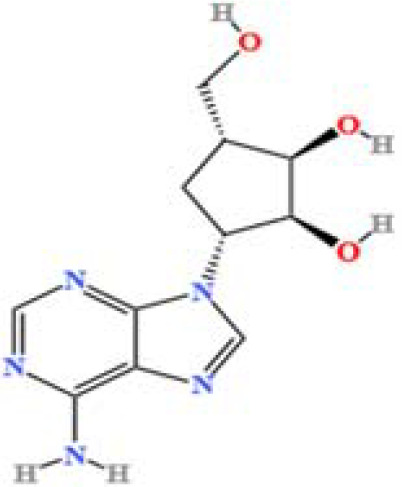
13	Trigonelline	C_7_H_7_NO_2_	581901381.210126	1.365	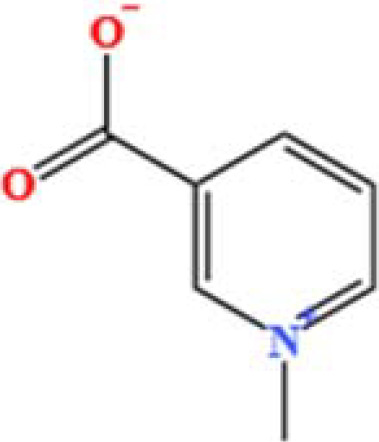
14	Daidzin	C_21_H_20_O_9_	579670874.883061	5.485	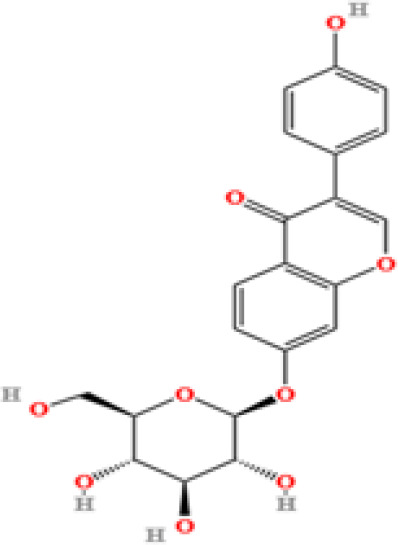
15	Acrylic acid	C_3_H_4_O_2_	400173626.5	1.655	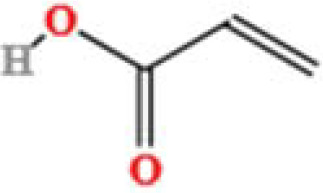
16	Daidzein	C_15_H_10_O_4_	353510052	7.626	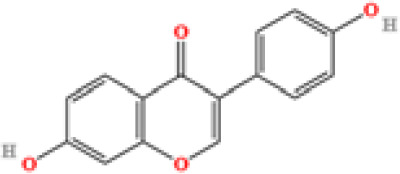
17	Cytosine	C_4_H_5_N_3_O	334570466.48315	1.587	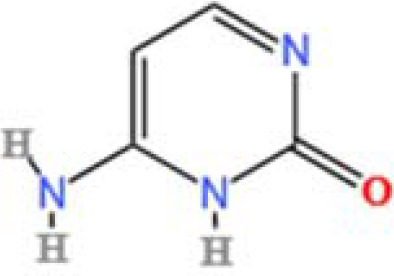
18	L-Pyroglutamic acid	C_5_H_7_NO3	241696305.2	1.316	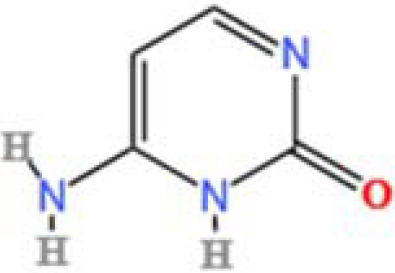
19	DL-Glutamine	C_5_H_10_N_2_O_3_	74694715.76	1.306	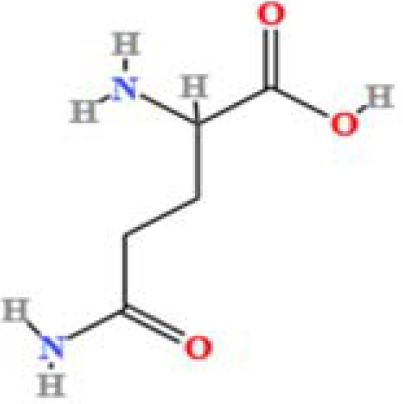
20	Cytidine	C_9_H_13_N_3_O_5_	31672205.7827671	1.586	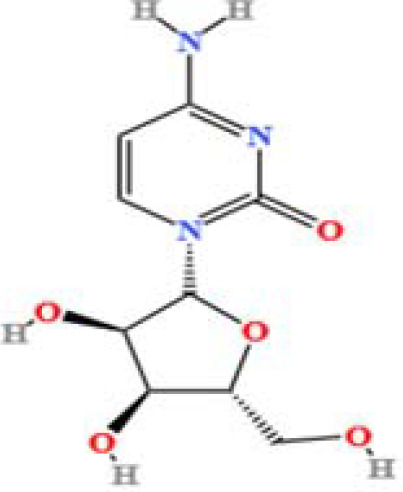
21	Urea	C H_4_N_2_O	23793971.4290239	1.36	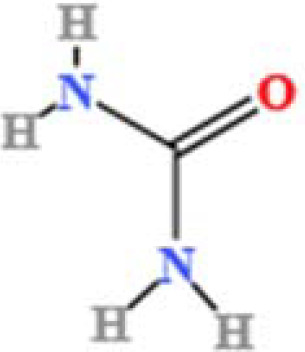
22	Acetylcholine	C_7_H_16_NO_2_^+^	8132478.83578916	1.626	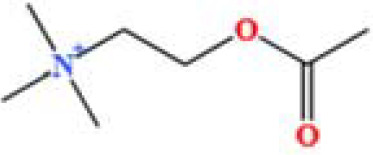

A total of 22 compounds were identified by comparing their retention times and mass spectra with those of reference substances. These compounds include Urea, Trigonelline, L-pyroglutamic acid, DL-glutamine, Cytosin, Daidzein, Cytidine, Betaine, Acetylcholine, Berberine, Ficine, L-(+)-Arginine, D-(+)-Proline, Choline, Puerarin, Indole-3-acrylic acid, Neplanocin A, Pipecolic acid, Aristeromycin, Terricollene B, Daidzin, Acrylic acid(**Table 2**).

### 3.2 Effect of SHTX on physiological and serological changes in diabetic rats

**Figure 2:**
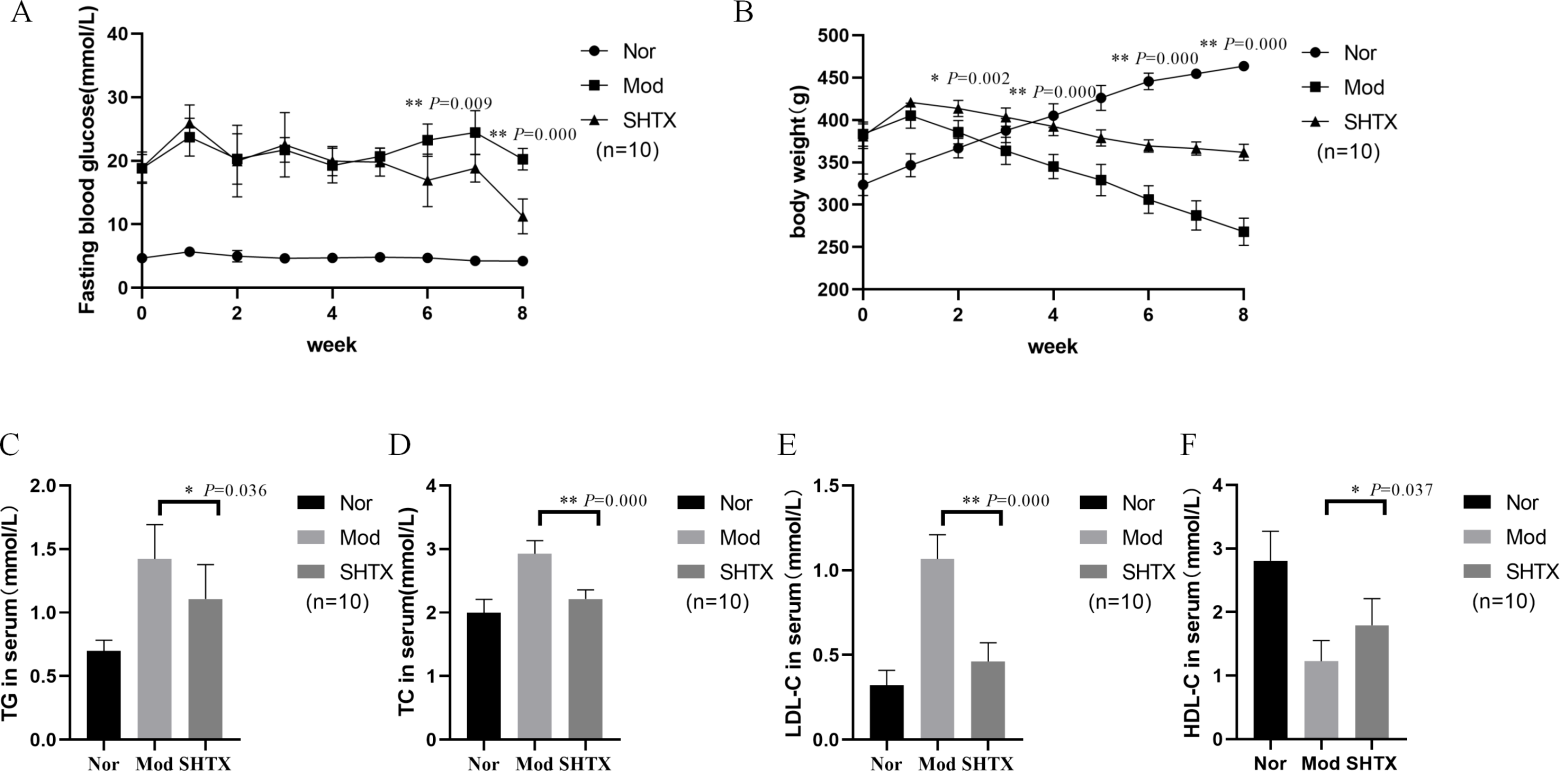
Effects of Sanghuangtongxie decoction (SHTX) on physiological and serological changes in rats with diabetic mellitus. **(A)** Fasting blood glucose (FBG). the SHTX group vs. the Mod group, 6th week, *P*=0.000. **(B)** Body weight. the SHTX group vs. the Mod group, 2th week, *P*=0.002. **(C)** Triglyceride (TG), the SHTX group vs. the Mod group, *P*=0.036. **(D)** Total cholesterol (TC), the SHTX group vs. the Mod group, *P*=0.000. **(E)** LDL-cholesterol (LDL-C), the SHTX group vs. the Mod group, *P*=0.000. **(F)** HDL-cholesterol (HDL-C), the SHTX group vs. the Mod group, *P*=0.037. Nor, normal group; Mod, STZ-induced T2DM model group; SHTX, SHTX 14g/kg treatment group. Data are expressed as the mean ± SD. (n=10 per group); Prior to statistical analysis, data normality was verified using the Shapiro–Wilk test, and homogeneity of variance was assessed via Levene's test. One-way analysis of variance (ANOVA) was employed to compare intergroup differences, followed by Tukey's post hoc test for multiple comparisons. All statistical tests were two-tailed, with analyses conducted using SPSS 25.0 and graphical representations were generated using GraphPad Prism 8.0.2. *P < 0.05 vs. the Mod group. **P < 0.01 vs. the Mod group.

At the start of the experiment, there were no significant differences in body weight and fasting blood glucose (FBG) among the control (Nor), model (Mod), and SHTX groups (*P* > 0.05). After 4 weeks of high-fat diet feeding, FBG and body weight in the Mod and SHTX groups were significantly higher than those in the Nor group (*P*< 0.01), while no significant differences were observed between the Mod and SHTX groups (*P* > 0.05), indicating that these two groups were comparable prior to treatment. During the 8-week experimental period, SHTX treatment at a dose of 14 g/kg significantly reduced fasting blood glucose (FBG) levels compared with the model group (**Figure 2A**), with statistical significance observed from week 6 onward (*P*< 0.01) and maintained through week 8. Additionally, body weight in the SHTX-treated group was significantly higher than that in the model group at week 2 (*P*< 0.01),and remained so until week 8 (**Figure 2B**) (**Tables 3**, **4**).

**Table 3:** Effect of SHTX on FBG in Rats( $\bar{\text{x}}$ ± *s*).

Group	n	FBG(mmol/L)				
		Before treatment	2 weeks after treatment	4 weeks after treatment	6 weeks after treatment	8 weeks after treatment
Nor	10	4.69±0.28	4.98±0.90	4.70±0.19	4.72±0.39	4.20±0.23
Mod	10	18.82±2.18	20.27±3.97	19.23±2.75	23.23±2.56	20.25±1.69
SHTX	10	18.90±2.49	19.95±5.64	19.93±2.32	16.92±4.13	11.23±2.74
*P*		0.952	0.913	0.644	0.009	0.000

**Table 4:** Effect of SHTX on Body Weight in Rats( $\bar{\text{x}}$ ± *s*).

Group	n	Weight(g)				
		Before treatment	2 weeks after treatment	4 weeks after treatment	6 weeks after treatment	8 weeks after treatment
Nor	10	323.67±12.68	366.83±11.58	405.00±14.06	445.67±9.79	463.67±4.76
Mod	10	383.34±14.25	385.67±13.91	345.17±14.34	306.17±16.29	268.17±16.02
SHTX	10	380.83±14.50	413.67±9.44	392.33±10.67	369.33±7.31	361.83±9.66
*P*		0.769	0.002	0.000	0.000	0.000

Regarding lipid analysis, SHTX treatment significantly reduced triglyceride (TG) levels (**Figure 2C**, *P*< 0.05) and total cholesterol (TC) (**Figure 2D**, *P*< 0.01), while low-density lipoprotein cholesterol (LDL-C) levels (**Figure 2E**) were significantly elevated (*P*< 0.01). Furthermore, high-density lipoprotein cholesterol (HDL-C) levels (**Figure 2F**) were significantly reduced in the SHTX group (*P*< 0.05), indicating that SHTX effectively ameliorates dyslipidemia in diabetic rats (**Table 5**).

**Table 5:** Effect of SHTX on TG, TC, LDL-C, HDL-C in Rats( $\bar{\text{x}}$ ± *s*).

Group	n	TG(mmol/L)	TC(mmol/L)	LDL-C(mmol/L)	HDL-C(mmol/L)
Nor	10	0.70±0.08395	1.99±0.21	0.32±0.088	2.81±0.46
Mod	10	1.42±0.27	2.93±0.21	1.07±0.14	1.23±0.32
SHTX	10	1.11±0.27	2.21±0.14	0.46±0.11	1.79±0.42
*P*		0.036	0.000	0.000	0.037

### 3.3 Effect of SHTX on histopathological changes in liver tissue of diabetic rats

**Figure 3:**
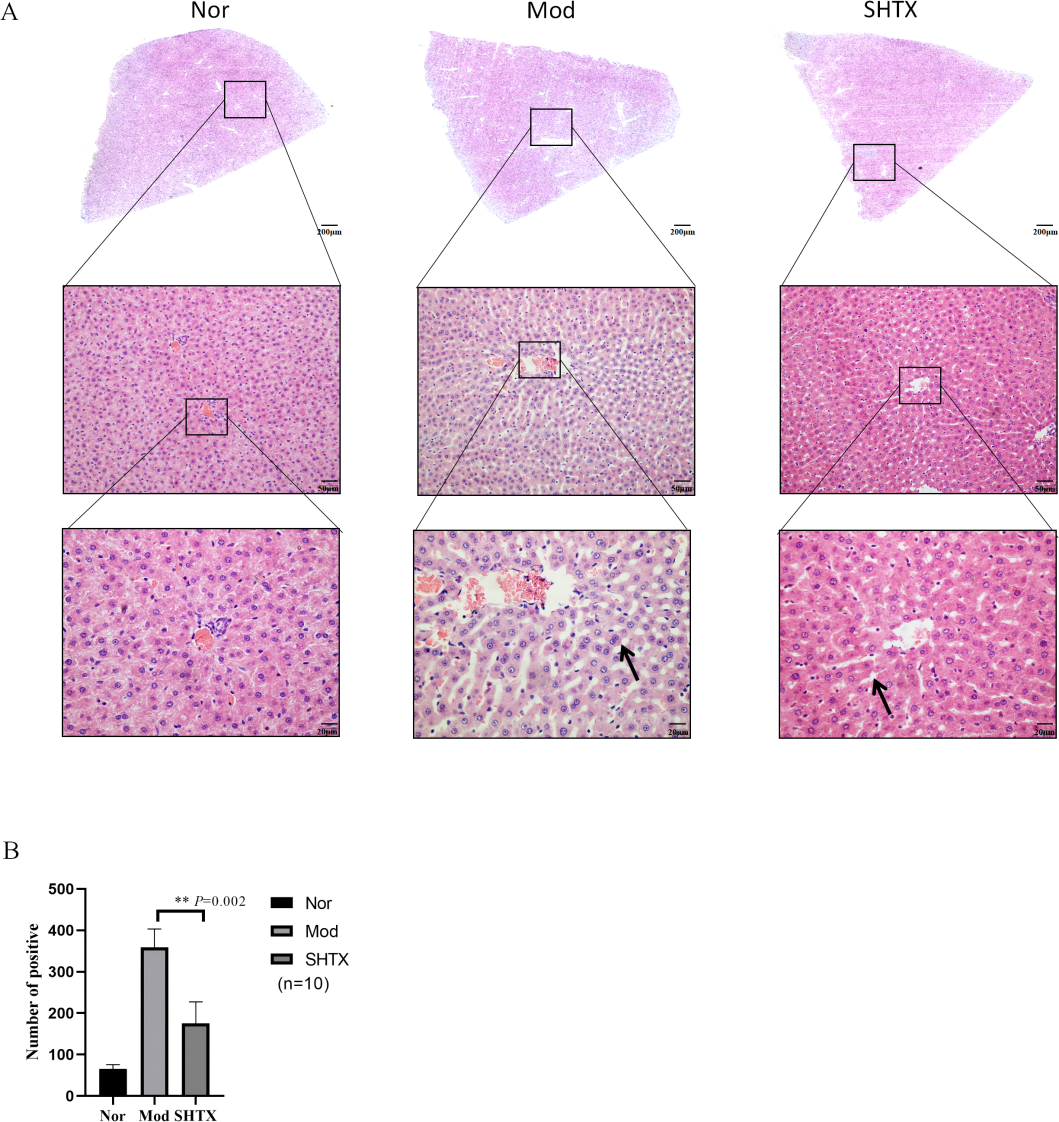
Following SHTX improved the pathological changes of liver in rats with type 2 diabetes mellitus. **(A)** H&E staining assay was explored to the changes of liver fibrous tissue. Nor: Hepatocytes radially arranged around central veins with normal morphology; Mod: Hepatocytes containing irregularly sized lipid droplets forming cord-like structures; SHTX group: marked reduction in steatosis, partial restoration of sinusoidal radial architecture, decreased lipid deposition. Three non-overlapping sections per rat, with five 200× fields randomly selected per section for lipid droplet counting; **(B)** Number of lipid droplets in liver sections. the SHTX group vs. the Mod group, *P*=0.037. Nor, normal group; Mod, STZ-induced T2DM model group; SHTX, SHTX 14g/kg treatment group. Data are expressed as the mean ± SD. (n=10 per group); One-way analysis of variance (ANOVA) was employed to compare intergroup differences, followed by Tukey's post hoc test for multiple comparisons. All statistical tests were two-tailed, with analyses conducted using SPSS 25.0 and graphical representations were generated using GraphPad Prism 8.0.2. **P < 0.01 vs. the Mod group.

To investigate the effects of SHTX on liver morphology in type 2 diabetic rats, liver tissue was examined using H&E staining. Compared with the model group, the number of lipid droplets was significantly reduced in the SHTX group (**Figure 3B**, *P*< 0.01). As shown in **Figure 3A**, liver sections from the normal group displayed hepatocytes with a well-organized radial arrangement centered around the central vein. In contrast, liver tissue from the model group showed clear signs of steatosis, with hepatocytes surrounding the central vein containing fat droplets of varying sizes, resulting in an irregular, cord-like structure. Following SHTX treatment, hepatic steatosis was markedly reduced, the radial architecture of the hepatic sinusoids was partially restored, and the accumulation of fat in hepatocytes was significantly diminished, leading to an improvement in the overall morphology of the liver tissue. All counts were performed by an experimenter unaware of the grouping. Three non-overlapping sections were analyzed per sample group. Lipid droplets were counted within five randomly selected high-power fields (200 x) per section using an optical microscope. The mean number of lipid droplets per high-power field per animal was calculated, with intergroup data expressed as mean ± SD.

### 3.4 SHTX improves mucosal barrier function

**Figure 4:**
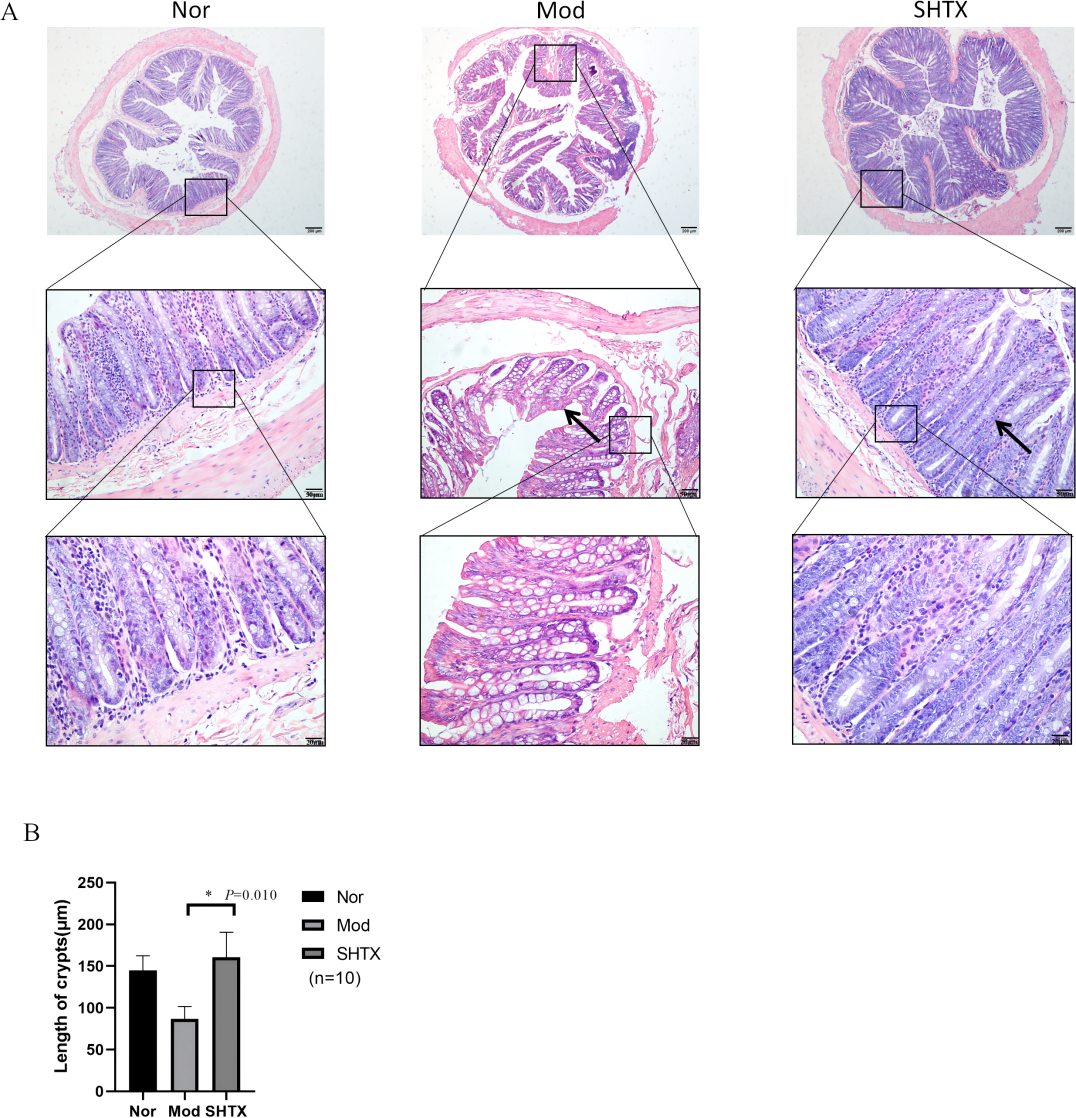
SHTX enhances colonic motility and improves mucosal barrier function. **(A)** Typical haematoxylin and eosin (H&E) stained colonic section. Normal: Colon mucosal epithelium intact with well-organised crypt architecture. Moderate: Mucosal epithelial damage with disrupted crypt architecture. SHTX group: Mucosal epithelial integrity restored with well-organised crypt architecture. **(B)** Quantitative analysis of crypt length. the SHTX group vs. the Mod group, *P*=0.010. Three non-overlapping sections per rat, with five 200× fields randomly selected per section for lipid droplet counting. Data are expressed as the mean ± SD. (n=10 per group); One-way analysis of variance (ANOVA) was employed to compare intergroup differences, followed by Tukey's post hoc test for multiple comparisons. All statistical tests were two-tailed, with analyses conducted using SPSS 25.0 and graphical representations were generated using GraphPad Prism 8.0.2. *P < 0.05 vs. the Mod group.

Hematoxylin and eosin (H&E) staining revealed significant epithelial barrier damage in the intestines of diabetic rats, including shortened and irregularly arranged crypts, epithelial cell detachment, and inflammatory cell infiltration. Quantitative analysis confirmed a significant reduction in crypt length in the model group compared with the normal group (**Figure 4B**, *P* < 0.05). In contrast, the SHTX-treated group exhibited marked improvement in intestinal morphology compared to the model group, suggesting that SHTX may exert a protective effect against diabetes-induced intestinal damage by restoring mucosal barrier function (**Figure 4A**). Histological assessment and crypt length measurement were conducted using the same blinded evaluation protocol, sample size, and section/field counting approach as described for liver tissue.

### 3.5 Impact of SHTX on the composition and structure of the gut microbiota

The homeostasis of the gut microbiota is characterized by the phyla *Firmicutes* and *Bacteroidetes*, which are composed of specialized anaerobic members. An increase in the relative abundance of Enterobacteriaceae (phylum Ascomycota) is commonly associated with gut dysbiosis. To assess the efficacy of Sanghuang Tongxie Formula (SHTX) in mitigating high-fat diet-induced gut microbiota dysbiosis, we examined the microbial composition of the gut in several rat groups using 16S rRNA gene amplicon sequencing in the caecum contents.

**Figure 5:**
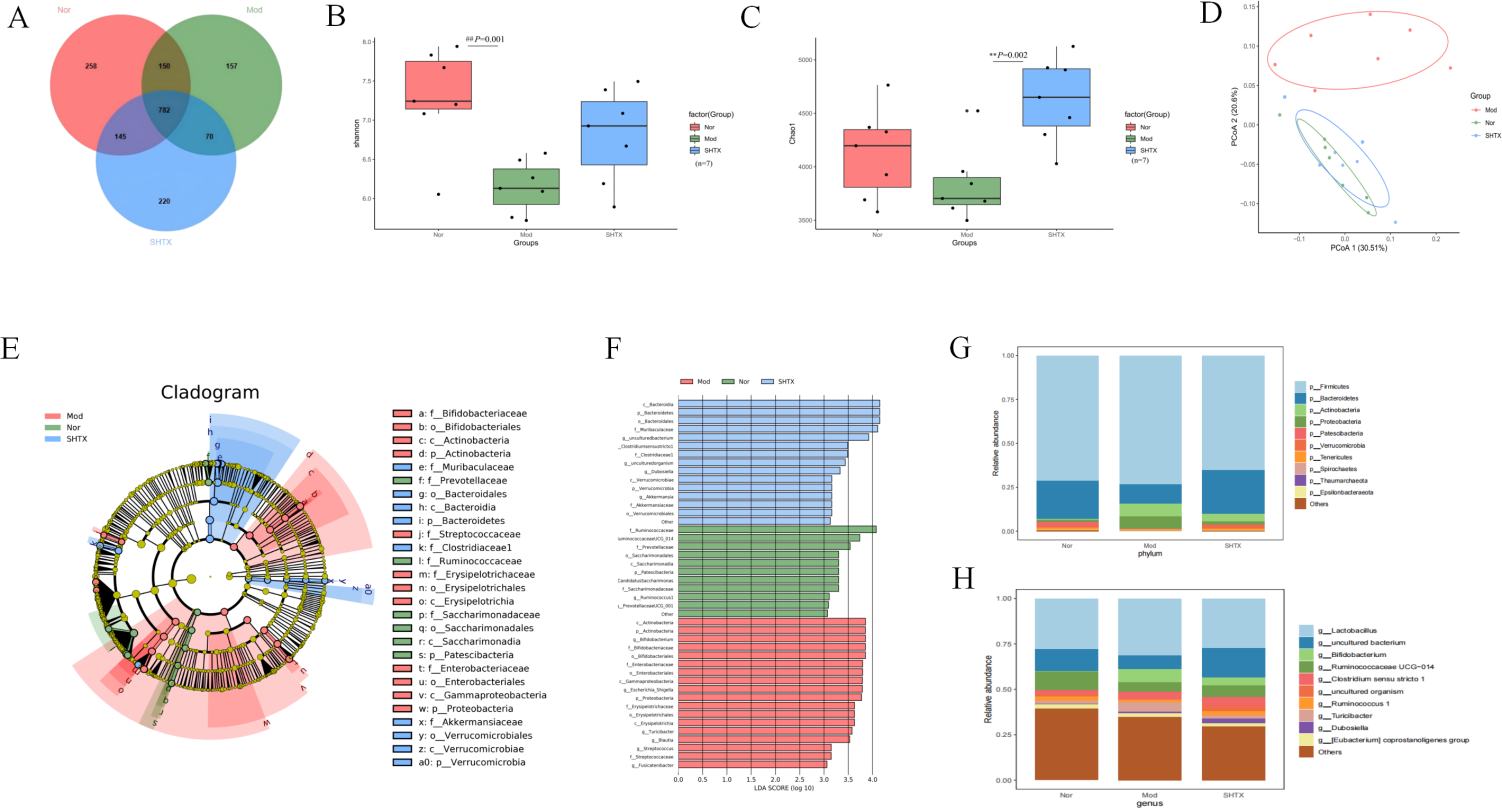
SHTX affect species diversity of gut microbiota in rats with T2DM. **(A)** Venn diagram of species richness. **(B)** Shannon indices of α-diversity. the Nor group vs. the Mod group, *P*=0.001. **(C)** Chao1 indices of α-diversity. the SHTX group vs. the Mod group, *P*=0.001. **(D)** PCoA using Weighted-UniFrad of beta diversity. **(E)** Taxonomic clade plots from LEfSe depicting taxonomic associations between microbial communities from the normal, model, and SHTX groups. Circles radiating inside to outside in the evolutionary clade map represent taxonomic levels from phyla to genus. Blue, green, and red nodes in the branches indicate microbial taxa that play important roles in the blue, green, and red groups, and yellow nodes indicate microbial taxa that did not play a significant role in both groups. **(F)** LDA scores based on features rich in differences between the three groups. The criterion for feature selection was LDA score > 3 and *P* < 0.05. SHTX administration alters the composition and structure of gut microbiota. **(G)** Bacterial community composition at phylum level. H.Bacterial community composition at genus level. (n=7 per group). Data are expressed as mean ± standard deviation. Intergroup differences were analysed using one-way analysis of variance. *P* < 0.05 was considered statistically significant. All statistical tests were two-tailed, with analyses conducted using SPSS 25.0, with graphical representations generated using R version 4.4.1. ^##^*P* < 0.05 the Nor group vs. the Mod group. **P < 0.01 the SHTX group vs. the Mod group.

Venn diagrams revealed that 782 operational taxonomic units (OTUs) were shared among all groups, while the normal, model, and SHTX-treated groups exhibited 1,040, 949, and 1,002 unique OTUs, respectively (**Figure 5A**). The Shannon index was used to assess microbial diversity (**Figure 5B**), while the Chao 1 index provided insights into the microbial richness (**Figure 5C**). Both the Chao 1 and ACE indices displayed significant differences across the groups, suggesting a reduction in both the quantity and diversity of the gut microbiota in the model group compared to the normal group. Notably, treatment with 14 g/kg SHTX significantly restored both the diversity and richness of the gut microbiota, as evidenced by the improved indices.

To further explore the impact of SHTX on microbial diversity, we performed beta diversity analysis using the Unweighted-UniFrac distance metric and conducted principal coordinate analysis (PCoA) (**Figure 5D**). Clear separation between the groups was observed, indicating significant heterogeneity in the gut microbiota composition. Linear discriminant analysis effect size (LEfSE) analysis of OTU abundance revealed distinctive microbiota profiles across the groups at various taxonomic levels (**Figures 5E, F**). LDA scores based on features rich in differences between the three groups. The criterion for feature selection was LDA score > 3 and *P* < 0.05.

At the phylum level (**Figure 5G**), we identified *Firmicutes* and *Bacteroidetes* as the predominant phyla across all groups, with their relative abundances exceeding 70% (12). SHTX treatment significantly increased the relative abundance of *Bacteroidetes* to the model group (*P* < 0.05). Additionally, the ratio of *Firmicutes* to *Bacteroidetes* (F/B ratio), a key marker of dysbiosis associated with diabetes and obesity, was significantly reduced in the SHTX group, suggesting a potential beneficial effect in restoring gut microbial balance (13, 14).

At the genus level (**Figure 5H**), we observed a significant increase in the relative abundance of *Lactobacillus* and *Bifidobacterium*, two major probiotics with anti-inflammatory properties, in the SHTX-treated group compared to the control group. Conversely, the relative abundances of *Turicibacter*, *Ruminococcaceae_UCG-014*, and *Ruminococcus 1* were significantly reduced in the model group (*P* < 0.05). These findings highlight the comprehensive effects of SHTX on the gut microbiota, suggesting its potential role in promoting the stability of beneficial bacteria and regulating dysbiosis.

### 3.6 Regulation of hepatic metabolic patterns in T2DM rats by SHTX

The gut microbiota exerts its effects on the host’s metabolic state through the production of metabolites, which can influence various tissues, including the liver (15). Blood metabolomics provides a comprehensive snapshot of these metabolic alterations, reflecting the functional changes in both the gut microbiota and host organs, thus facilitating inter-organ signaling. This study employed metabolomics to analyze liver samples from T2DM rats, aiming to elucidate the potential mechanisms through which Sanghuang Tongxie Formula (SHTX) modulates hepatic metabolism, using UHPLC-Q-TOF/MS to acquire total ion chromatograms in both positive and negative ion modes. Following data preprocessing (peak alignment, denoising, normalization), a total of 697 annotatable metabolites were identified. Among these, 297 annotatable metabolites were identified in positive ion mode, while 400 annotatable metabolites were identified in negative ion mode.

**Figure 6:**
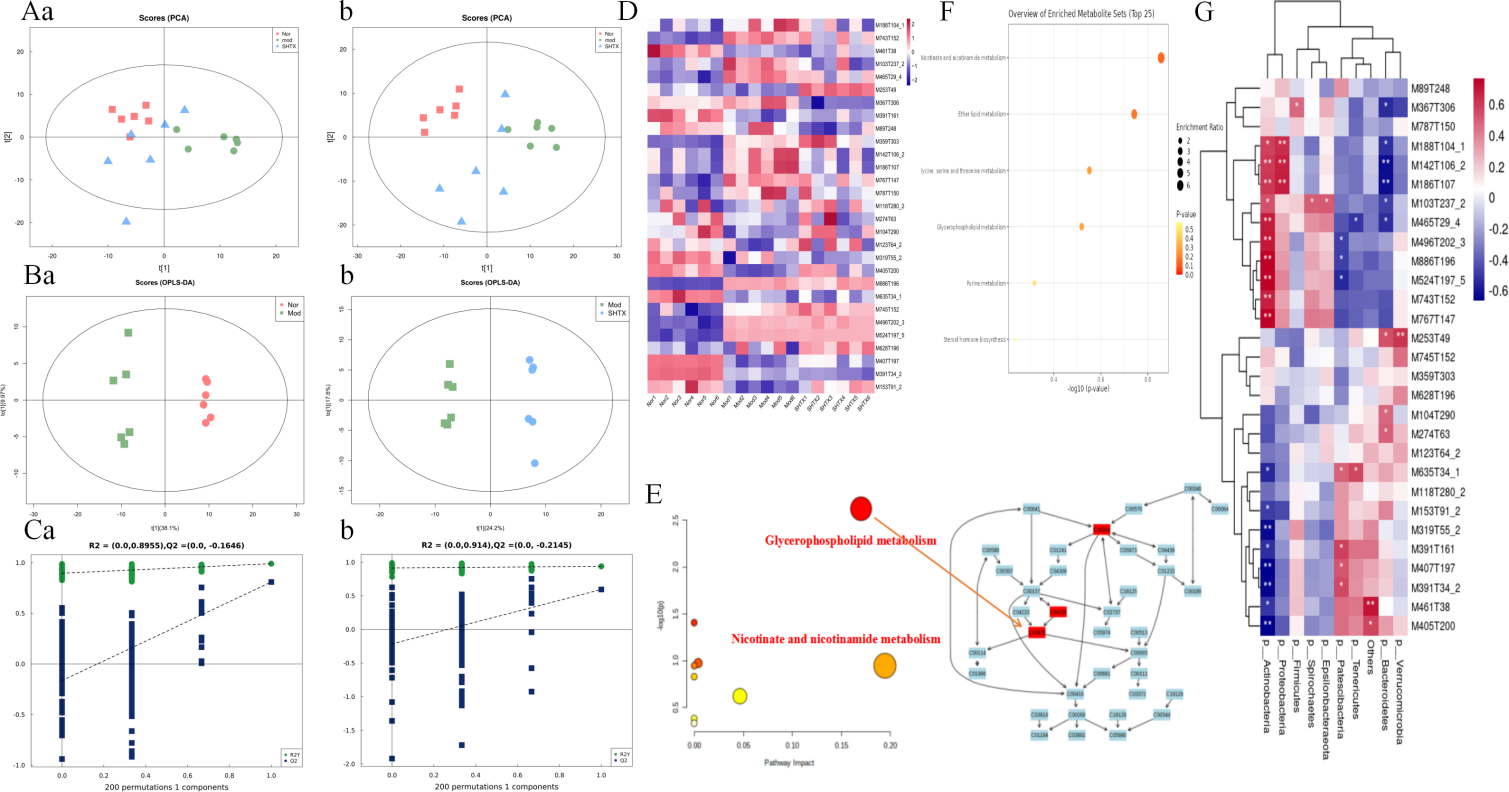
Sanghuang Tongxie Formula (SHTX) regulates hepatic non-targeted metabolomic profiles in type 2 diabetic (T2DM) rats. **(A)** The PCA score plots for the negative ion mode **(a)** and in the positive ion mode **(b)** from the Normal, model, SHTX groups. **(B)** OPLS-DA score plot for the Nor group and Mod group **(a)**. OPLS-DA score plot for the SHTX group and Mod group **(b)**. **(C)** Permutation test results for the OPLS-DA models of the Nor group and Mod group **(a)**. Permutation test results for the OPLS-DA models of theSHTX group and Mod group **(b)**. **(D)** Heat map analysis of 29 differential metabolites identified between Nor, Mod, and SHTX groups. The screening criteria were VIP > 1 and FDR-adjusted P < 0.05. **(E)** Each colored spot represents a metabolic pathway, Color gradient represents the p-value size (red: higher p-values and blue: lower p-values), and circle size represents the rank of pathway impact score (the larger the circle: higher impact score, and the smaller the circle: lower impact score).**(F)** Each bubble in the bubble diagram represents a metabolic pathway. The abscissa and the bubble size indicate the influence factor of the pathway in the topology analysis. The larger the size, the larger the influence factor; the bubble ordinate and bubble color indicate the p-value of the enrichment analysis, the darker the p-value, the more significant the enrichment degree. pathway enrichment analyzed using MetaboAnalyst with *P* < 0.05 as significant. **(G)** Correlation diagram of gut bacteria and metabolites. (n=6 per group). Plotting was performed using R4.4.1 and Image GP software.

Principal component analysis (PCA) was performed to assess the metabolite profiles of liver samples across the different experimental groups. The results revealed distinct clustering of metabolites in both the positive ion mode (**Figure 6Aa**) and negative ion mode (**Figure 6Ab**), indicating significant alterations in the liver metabolic profiles among the treatment groups. PCA clearly distinguished the metabolic profiles of the normal and model groups, with the SHTX-treated group showing a trend toward normalization, suggesting that SHTX has a regulatory effect on hepatic metabolism.

To further validate the observed metabolomic differences, Orthogonal Partial Least Squares Discriminant Analysis (OPLS-DA) was employed for supervised multivariate statistical analysis. The OPLS-DA score plots demonstrated high R²Y and Q² values (Nor vs. Mod, R²Y = 0.99, Q² = 0.907; Mod vs. SHTX, R²Y = 0.997, Q² = 0.799), indicating excellent model fit and predictive capacity (**Figures 6Ba, b**). The R² values approaching 1 reflect the robustness of the model, while Q² values exceeding 0.5 underscore its strong predictive power. In addition, 200-times permutation testing was performed to assess potential overfitting, and the results showed that the permuted R² and Q² values were significantly lower than the original values, confirming the reliability and validity of the OPLS-DA models (**Figures 6Ca, b**).

Significant differential metabolites were identified based on VIP > 1, FDR-adjusted *P* < 0.05, resulting in the identification of 29 annotated metabolites (**Supplementary Table 1**). To visualize these differences, heatmap analysis was conducted using Image GP software (**Figure 6D**), which revealed marked disparities in metabolite abundance across the groups. In the model group, the concentrations of Nicotinamide, Glycerophosphorylcholine, and Xanthine were significantly elevated, while metabolites such as Beta-hydroxybutyric acid, Cholesteryl sulfate, Aminoacrylic acid, and indolactic acid were markedly reduced. In contrast, the metabolite expression profile in the SHTX group closely resembled that of the normal group, suggesting that SHTX effectively modulated hepatic metabolism in T2DM rats.

To explore the metabolic pathways affected by SHTX, pathway enrichment analysis was performed on the significantly altered metabolites using the MetabolAnalyst platform (https://www.metaboanalyst.ca/) (**Figure 6E**). The analysis identified six major metabolic pathways associated with SHTX treatment (**Figure 6F**), including Nicotinic acid and nicotinamide metabolism, Ether lipid metabolism, Glycine, serine and threonine metabolism, Glycerophospholipid metabolism, Purine metabolism, and Steroid hormone biosynthesis. Notably, glycerophospholipid metabolism emerged as a particularly significant pathway, underscoring its potential role in the antidiabetic effects of SHTX.

To investigate the interactions between the gut microbiota and metabolism, we conducted association analyses and presented the results as a heatmap (**Figure 6G**). The results indicate that p_Actinobacteria exhibits a positive correlation with1h-indole-3-propanoic acid(ID: M188T104_1), Indoleacrylic acid(ID: M142T106_2), Indolelactic acid(ID: M186T107), Beta-hydroxybutyrate(ID: M103T237_2), Cholesteryl sulfate(ID: M465T29_4), Pi 38:4(ID: M886T196), 1,2-dioleoyl-sn-glycero-3-phosphoethanolamine(ID: M745T152) and a negative correlation with 1,2-dilinoleoylglycerol(ID: M635T34_1), Xanthosine(id: M153T91_2),12s-hydroxy-5z,8z,10e,14z-eicosatetraenoic acid(ID: M319T55_2), Deoxycholic acid(ID: M391T161), Dioctyl phthalate(ID: M391T34_2), Arachidonoylserotonin(ID: M461T38),3-dehydrocholic acid(ID: M405T200). p_Proteobacteria exhibits a positive correlation with 1h-indole-3-propanoic acid, Indoleacrylic acid, Indolelactic acid. p_Firmicutes exhibits a positive correlation with Curcumin (ID: M367T306). p_Bacteroidete exhibits a positive correlation with Curcumin, 1h-indole-3-propanoic acid, Indoleacrylic acid, Indolelactic acid, Beta-hydroxybutyrate, Cholesteryl sulfate and a negative correlation with Cis-9-palmitoleic acid(ID: M253T49), Glycerophosphocholine(ID: M104T290), Fenpropidin(ID: M274T63).

### 3.7 Regulation of hepatic gene expression patterns by SHTX

**Figure 7:**
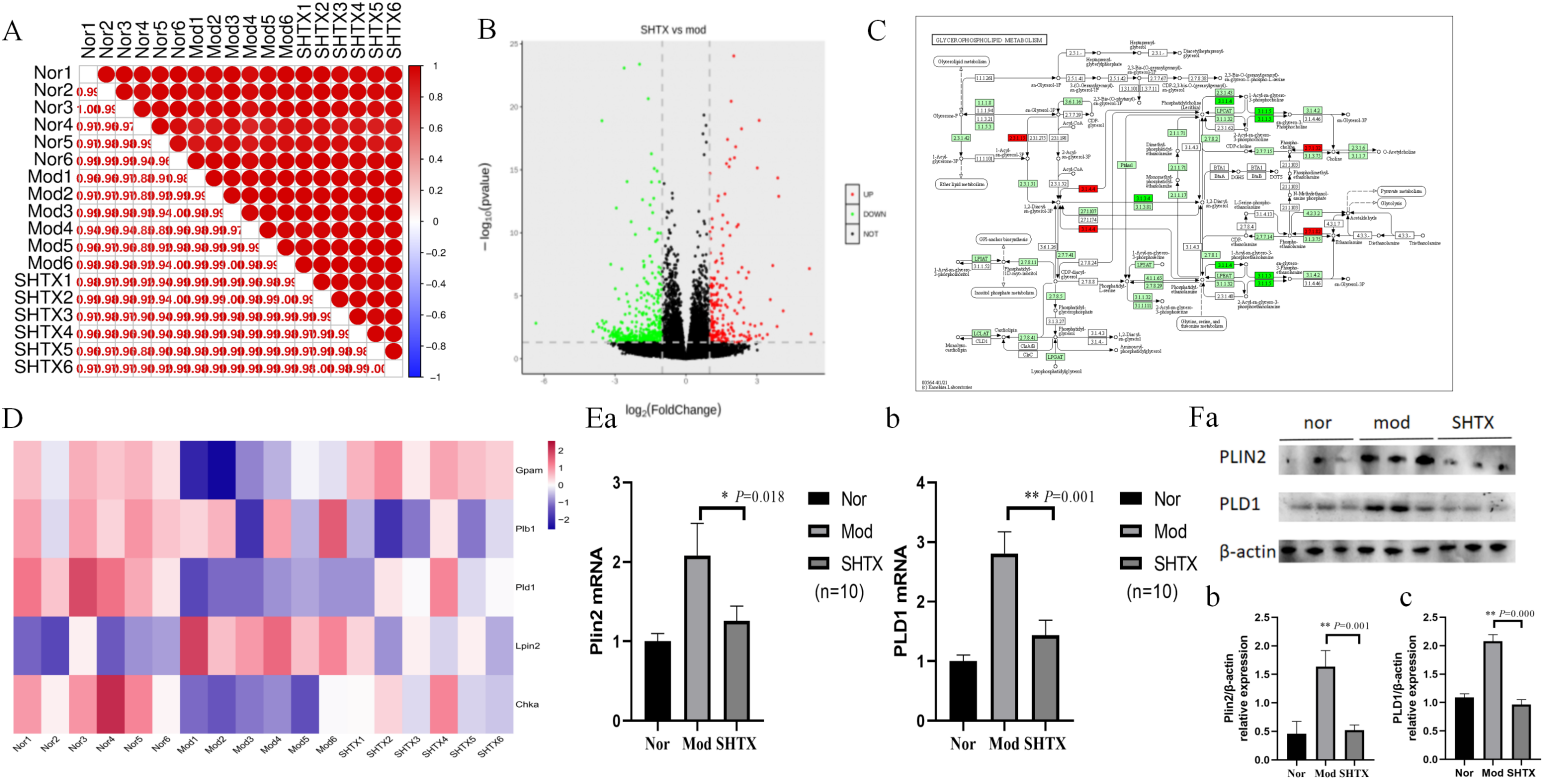
Sanghuang Tongxie Formula (SHTX) regulates hepatic gene expression and protein levels related to glycerophospholipid metabolism in type 2 diabetic (T2DM) rats. **(A)** Heat map of the replicate samples of the Normal, model, SHTX groups. (n=6 per group). The color spectrum, ranging from red to blue, represents Pearson’s correlation coefficients ranging from 1 to 0.975, indicating high to low correlations. **(B)** The Volcano plot showing the relationship between the fold-change (on the X-axis) and the significance of the differential expression test (Y-axis) for each gene from DESeq_2_ analysis. The screening criteria were |log2FC| > 1and P-value < 0.05. Black dots represent the genes that are not significantly differentially expressed, while red and green dots are the genes that are significantly up- and down-regulated, respectively. **(C)** The light green background shows the genes in which the species genome exists in the KEGG pathway; red background indicates the differential genes involved in the pathway, dark green indicates the downregulated genes involved in the pathway; small circles represent small molecule metabolites, and large circular boxes represent other pathways. **(D)** Heat map of differentially expressed genes in glycerophospholipid metabolism. Genes with |log_2_FC| > 1 and FDR < 0.05 were considered to be differentially expressed. Red and Blue in the colour bar represent high and low expression levels, respectively. **(E)** The levels of Plin2 and PLD1(b) mRNAs were detected by the qRT-PCR assay. Plin2(a), the SHTX group vs. the Mod group, *P*=0.018. PLD1(b), the SHTX group vs. the Mod group, *P*=0.001. (n=10 per group). **(F)** Immunoblot expression of Plin2 and PLD1 proteins from liver and their quantification normalized with β-actin control. Plin2(b), the SHTX group vs. the Mod group, *P*=0.001. PLD1(c), the SHTX group vs. the Mod group, *P*=0.000. Nor, normal group; Mod, STZ-induced T2DM model group; SHTX, SHTX 14g/kg treatment group. Data are expressed as the mean ± SD. (n=10 per group); One-way analysis of variance (ANOVA) was employed to compare intergroup differences, followed by Tukey's post hoc test for multiple comparisons. All statistical tests were two-tailed, with analyses conducted using SPSS 25.0 and graphical representations were generated using GraphPad Prism 8.0.2. *P < 0.05 vs. the Mod group. **P < 0.01 vs. the Mod group.

This study performed a comprehensive genome-wide transcriptional analysis of rat liver tissues using RNA sequencing to explore the relationship between the previously identified metabolite profiles and the transcriptional activity of specific genes. Pearson correlation analysis (**Figure 7A**) revealed a robust correlation among all 18 samples, confirming the consistency and reliability of the samples within each treatment group. Subsequent analysis uncovered significant transcriptomic differences between the SHTX-treated and control rats (**Figure 7B**). Using the criteria of |log_2_FC| > 1and a P-value < 0.05, we identified 641 differentially expressed genes, including 229 up-regulated and 412 down-regulated genes in the liver of the SHTX-treated group.

Genes associated with glycerophospholipid metabolism were then extracted by matching DEGs to the KEGG glycerophospholipid metabolism pathway (KEGG: map00564), resulting in 95 candidate genes (**Figure 7C**). Among these, 11 genes were up-regulated, while 29 were down-regulated in response to SHTX treatment. Notably, SHTX treatment significantly restored the expression of five key genes involved in lipid metabolism, including Plb1, Gpam, Pld1, Chka and Lpin2. Heatmap analysis (**Figure 7D**) demonstrated marked differences in gene expression between the SHTX and model groups, with the gene expression profile of the SHTX group closely resembling that of the normal group.

To further validate the transcriptional changes, we performed real-time quantitative PCR (RT-qPCR) to assess the mRNA levels of key genes involved in glycerophospholipid metabolism (**Figure 7E**). The results demonstrated that Plin2 expression levels were significantly reduced in the SHTX-treated group compared with the model group (**Figure 7Ea**, *P* < 0.05). Furthermore, Pld1 expression levels also exhibited a marked downregulation trend in the SHTX-treated group (**Figure 7Eb**, *P* < 0.01).

These findings suggest that SHTX treatment markedly modulates the expression of genes associated with glycerophospholipid metabolism. Additionally, protein expression of Pld1 and Plin2 was assessed via Western blot analysis (**Figure 7Fa**). Compared with the model group, expression of the Plin2 and Pld1 proteins was significantly reduced in the SHTX group (**Figures 7Fb,c**, *P* < 0.01), further supporting the transcriptional data.

## 4 Discussion

Type 2 Diabetes Mellitus (T2DM) is a complex endocrine and metabolic disorder characterized by chronic hyperglycemia and impaired insulin sensitivity (16). Clinically, it is typically associated with symptoms such as polydipsia, polyphagia, polyuria, and weight loss. Numerous studies have demonstrated that factors including dysregulated lipid metabolism, chronic inflammation, and oxidative stress are crucial in the onset and progression of T2DM (17–19).

SHTX is a traditional Chinese medicinal formulation composed of several herbs, including Sang Bai Pi, Huang Lian, Hou Pu, Zhi Mu, Shan Zhu, and other ingredients. LC-MS analysis in this study identified 22 biologically active compounds in SHTX, including Berberine, Puerarin, Trigonelline, L-pyroglutamic acid, DL-glutamine, and Indole-3-acrylic acid. Berberine has been shown to significantly improve insulin resistance and exhibit both hypoglycemic and hypolipidemic effects (20). Meanwhile, research by Liu et al. indicates that in the Goto-Kakizaki (GK) rat model of T2DM, berberine significantly improves glucose metabolism by regulating the gut microbiota. It demonstrates marked effects in enhancing metabolic parameters and reshaping the composition of the intestinal microbiome (21). Puerarin has demonstrated the ability to improve the overall physiological state of diabetic rats, significantly reducing blood glucose and HbA1c levels (22). Trigonelline has been shown to inhibit the function of glucose transporter protein 4 (GLUT4) by inducing the expression of peroxisome proliferator-activated receptor gamma (PPAR-γ), thereby improving glucose metabolism (23). Overall, SHTX exerts its antidiabetic effects through multiple pathways, including the regulation of glucose and lipid metabolism, reduction of oxidative stress, modulation of gut microbiota composition, and improvement of insulin sensitivity. However, further investigation into its specific molecular mechanisms remains essential for optimizing its therapeutic potential and clinical application.

In this study, we systematically evaluated the antidiabetic mechanisms of SHTX using a diabetic rat model. Our findings indicate that SHTX significantly reduced blood glucose levels and promoted body weight recovery in diabetic rats. Dysregulated lipid metabolism is a hallmark of T2DM, and the disease is often associated with enhanced lipolysis, leading to elevated serum levels of total cholesterol (TC) and triglycerides (TG) (24). Our study demonstrated that SHTX significantly reduced serum levels of TC, TG, and low-density lipoprotein cholesterol (LDL-C), while simultaneously increasing high-density lipoprotein cholesterol (HDL-C) levels in diabetic rats. Given that LDL-C is the primary carrier of endogenous cholesterol and elevated levels are associated with an increased risk of hypercholesterolemia, and that HDL-C facilitates cholesterol transport and elimination, the elevation of HDL-C levels positively influences the improvement of dyslipidemia and further supports body weight recovery (25, 26).

Histopathological findings revealed that in the liver, SHTX treatment significantly reduced lipid droplet accumulation and partially restored the radial arrangement of hepatic sinusoids, suggesting that SHTX may alleviate lipid metabolism disorders in type 2 diabetes by restoring hepatocyte morphology and function. In the intestine, the marked recovery of crypt length and epithelial integrity following SHTX administration indicated enhanced mucosal barrier function. Maintaining intestinal barrier integrity limits endotoxin translocation and systemic inflammation, thereby promoting improved metabolic homeostasis. Thus, SHTX ameliorates diabetes-induced hepatic and intestinal injury.

The gut-liver axis plays a pivotal role in the pathophysiology of T2DM. The liver-gut axis regulates metabolic homeostasis through bidirectional crosstalk, with microbial antigens, metabolites, and bile acids serving as key mediators. This axis modulates lipid metabolism in both the gut and liver while simultaneously shaping the structure and function of the gut microbiota. Disruption of gut microbiota composition and intestinal barrier integrity is increasingly recognized as a driver of lipid metabolism disorders and systemic insulin resistance (27, 28). In this study, 16S rRNA gene sequencing analysis revealed that the SHTX treatment significantly enhanced the richness and diversity of the gut microbiota at the OTU level. Numerous studies demonstrated that the richness and diversity of gut microbiota are reduced in T2DM rats, and increasing them is good for the treatment of T2DM (29, 30). The *Bacteroidetes* and *Firmicutes* are predominant in both rat and human gut microbiota. In our study, although the *Firmicutes* was dominant across all groups, its relative abundance was significantly reduced in the SHTX-treated group, and the Firmicutes/Bacteroidetes ratio was also markedly decreased. This finding is consistent with previous research. A study on obese type 2 diabetic mice revealed marked gut microbiota dysbiosis, where the Firmicutes/Bacteroidetes (F/B) ratio in the T2DM model group was closely associated with the pathological progression of obesity and T2DM (31). Another research indicates that Nogo-B deficiency reduces the F/B ratio, concurrently with the alleviation of hyperlipidemia, improvement in hepatic injury, and restoration of hepatic biosynthetic capacity. This suggests that alterations in gut microbiota composition may regulate lipid metabolism by influencing liver function (32). In addition to the F/B ratio, the abundance of specific bacterial genera has been implicated in metabolic regulation. Beyond the F/B ratio, the abundance of specific bacterial genera is also recognized as being associated with metabolic regulation, such as *Bifidobacterium*. *Bifidobacteria* can ferment indigestible carbohydrates to produce short-chain fatty acids (SCFAs), which promote glucagon-like peptide-1 (GLP-1) secretion, enhance insulin sensitivity, regulate hepatic lipid synthesis, and alleviate inflammation. Research indicates that increased *bifidobacteria* in red beans correlates closely with reduced plasma triglycerides, alleviated obesity, improved glucose homeostasis, and diminished hepatic steatosis (33).

SHTX treatment also led to a substantial decrease in the relative abundance of *Turicibacter*, a genus within the phylum *Firmicutes*, with *Turicibacter* sanguinis identified as the only species (34). Previous studies have demonstrated that colonization by T. sanguinis significantly alters intestinal lipid metabolism, lowers systemic triglyceride levels, and impacts the physiological condition of white adipose tissue in mice (35). This suggests a potential link between *Turicibacter* and metabolic irregularities in T2DM (36). Therefore, these alterations in gut microbiota composition provide a plausible rationale for the metabolic improvements observed following SHTX treatment.

Metabolome profiling further confirms the role of the gut-liver axis in SHTX treatment. Using an untargeted metabolomics approach coupled with UPLC-Q-TOF/MS (Ultra Performance Liquid Chromatography Quadrupole-Time of Flight Mass Spectrometry), we identified 29 distinct hepatic metabolites across three rat cohorts: normal, model, and SHTX-treated. These metabolites included glycerophosphorylcholine (GPC), glycerophosphoethanolamine (GPE), amino acids and peptides, purine metabolites, among others. Notably, phospholipid metabolites such as choline, glycerophosphoethanolamine, and glycerophosphorylcholine displayed significant variation across groups. GPC and GPE are key intermediates in glycerophospholipid metabolism, participating in membrane structure maintenance and lipid signaling regulation (37). In mammals, glycerophospholipids, such as phosphatidylcholine (PC) and phosphatidylethanolamine (PE), are the primary analogs (38). These lipids are integral components of cell membranes and play a crucial role in regulating hepatic lipid metabolism. They are closely linked to the pathophysiology of metabolic disorders, including insulin resistance and diabetes mellitus (39, 40). Nolan et al. have suggested that disruptions in glycerophospholipid metabolism may impair both carbohydrate and lipid metabolism by affecting insulin secretion (41). Nicotinamide, a metabolite derived from nicotinic acid via amidation, is involved in numerous physiological processes, including glucose glycolysis, lipid metabolism, and pyruvate metabolism (42).

In this study, we found that the concentrations of nicotinamide, glycerophosphorylcholine, and xanthine were significantly lower in the model group of diabetic rats, but these metabolite levels markedly increased following treatment with SHTX. Nicotinamide serves as a key precursor for NAD^+^ biosynthesis in mammals, mediating relevant metabolic pathways via the rate-limiting enzyme NAMPT. This pathway becomes impaired under high-fat dietary conditions, subsequently disrupting glucose homeostasis and impairing the function of NAD^+^-dependent proteins such as SIRT1. As a nicotinamide derivative, nicotinamide mononucleotide (NMN) can restore NAD^+^ levels in high-fat diet- or age-induced type 2 diabetic mice, thereby improving their impaired glucose tolerance and enhancing hepatic insulin sensitivity (43). Glycerophosphocholine (GPC), a choline-containing glycerophospholipid degradation product, is hydrolyzed by pancreatic phospholipase A in the intestinal lumen to form 1-acylglycerophosphocholine. Upon entering mucosal cells, it undergoes partial reacylation to form phosphatidylcholine, while the remainder is further hydrolyzed into glycerophosphocholine, glycerophosphate, glycerol, and phosphoethanolamine P (i). Subsequently, fatty acids and glycerophosphate are reassembled via the Kennedy pathway to generate triacylglycerol (44). Adequate GPC levels facilitate normal lipoprotein assembly and secretion, whereas metabolic syndrome frequently disrupts this process. Xanthine, an intermediate in purine metabolism, yields reactive oxygen species upon oxidation by xanthine oxidase; alterations in its levels reflect changes in nucleotide turnover and redox balance, both of which correlate with insulin resistance (45).

The synchronized alterations in gut microbiota composition suggest that microbial metabolites may have facilitated these hepatic metabolic shifts. Increased bifidobacteria promote short-chain fatty acid (SCFA) production and modulate bile acid profiles, both of which activate hepatic AMPK and PPAR signaling pathways (46, 47). This activation enhances glycerophospholipid metabolism and upregulates NAD^+^ synthesis pathways, potentially explaining the restoration of nicotinamide and GPC levels (48, 49). However, this inference is based solely on correlational evidence and requires further experimental validation. Notably, similar metabolic regulation mechanisms via the liver-gut axis have been observed in studies of other herbal active components. A comprehensive multi-omics study confirmed that dietary alpha-linolenic acid (ALA) enhances intestinal barrier integrity, reduces systemic lipopolysaccharide levels, and remodels the gut microbiota in db/db mice and STZ/NA-induced diabetic rat models. Metagenomic and metabolomic analyses revealed increased short-chain fatty acid (SCFA) production, accompanied by alterations in plasma metabolic profiles.

Disruption of glycerophospholipid homeostasis has been demonstrated to be a key contributor to insulin resistance and dyslipidemia in type 2 diabetes mellitus (T2DM). We focus on the key genes Plin2 and PLD1 within the glycerophospholipid metabolic pathway. Western blotting and quantitative real-time PCR results consistently indicate that the expression levels of enzymes involved in glycerophospholipid metabolism (such as Plin2 and PLD1) were markedly aberrant in T2DM rats, indicating a substantial disruption of glycerophospholipid metabolism. Following SHTX treatment, the expression levels of both genes were significantly reduced to levels close to those in the normal control group. Perilipin 2 (Plin2), a protein located on the surface of lipid droplets, plays a key role in stabilizing these droplets and facilitating the release of fatty acids from adipocytes (50). McManaman et al. (51) showed that deletion of Plin2 significantly reduced obesity symptoms in both male and female mice subjected to a high-fat diet, highlighting the critical role of Plin2 in lipid metabolism.

Plin2, a critical adipogenic gene, was found to have significantly lower expression in the livers of diabetic rats following stimulation with Porphyromonas gingivalis (P. gingivalis) in the study by Yoshida et al (52). This reduction in Plin2 expression led to disturbances in lipid homeostasis. The synthesis and degradation of glycerophospholipids, such as phosphatidylcholine (PC) and phosphatidylethanolamine (PE), are tightly regulated in the liver, with phospholipases playing an essential role in the degradation of these phospholipids (53). The regulation of Plin2 and PLD1 expression by SHTX in this study may restore the balance between glycerophospholipid synthesis and degradation by influencing phospholipase activity in the liver. This provides a genetic-level explanation for its improvement of lipid metabolism abnormalities in T2DM rats.

## 5 Study limitations

Although this study presents promising preliminary findings regarding the therapeutic potential of Sanghuang Tongxie Formula (SHTX) for type 2 diabetes mellitus (T2DM), several limitations remain. First, while SHTX demonstrated substantial efficacy in animal models findings cannot be directly extrapolated to human trials due to species differences in metabolic regulation, gut microbiota composition, and disease pathophysiology. To address this, future studies will focus on typical clinical cohorts of type 2 diabetes, prioritizing efficacy assessment, mechanism-of-action analysis, and safety evaluation. Follow-up periods will be extended to assess the durability and consistency of SHTX’s therapeutic effects.

Second, although SHTX treatment significantly influenced the gut microbiota, metabolome, and transcriptome, the precise mechanisms underlying these alterations remain unclear. The causal relationship between the Mycobacterium phylum and glycerophospholipid metabolism is yet to be fully elucidated. Subsequent studies will further investigate the relationship between gut microbiota/metabolite changes and the regulation of hepatic glycerophospholipid metabolism through experimental methods such as fecal microbiota transplantation and metabolite intervention, providing additional experimental evidence for the mechanism by which SHTX improves T2DM.

Moreover, a limitation of this study lies in the absence of a positive control group treated with conventional therapies (e.g., metformin).This gap prevents us from benchmarking the efficacy of SHTX against conventional protocols, making it difficult to contextualize the relative advantages or characteristics of SHTX. Future research will incorporate a positive control group receiving standard conventional treatment (e.g., metformin).

Finally, although the sample size of the animal models in this study was sufficient to meet the statistical significance requirements for this specific design, subsequent research plans aim to expand the animal sample size while selecting animal models from different strains.

## 6 Conclusion

In conclusion, while our study provides initial evidence supporting the efficacy of SHTX in the treatment of T2DM, further extensive research is required to clarify its mechanisms of action and assess its therapeutic applicability. In future investigations, we will aim to develop and validate personalized therapeutic strategies tailored to individual gut microbiome and metabolic profiles, with the goal of optimizing SHTX treatment efficacy in T2DM. This approach will leverage the integrated analysis of gut microbiota, metabolomic, and transcriptomic data to construct patient-specific response models, thereby providing a more comprehensive mechanistic understanding of SHTX’s effects and facilitating its translation into precision medicine applications.

## Data Availability

The datasets presented in this study can be found in online repositories. The names of the repository/repositories and accession number(s) can be found in the article/**Supplementary Material**.
