# Supplementary figures and images for: Multi level effects of Sanghuang Tongxie Formula on type 2 diabetes rats: a comprehensive analysis from intestinal bacteria to metabolome and transcriptome
Source: Front Endocrinol (Lausanne). 2025 Nov 11;16:1562105. doi: 10.3389/fendo.2025.1562105 (PMC12643869; doi:10.3389/fendo.2025.1562105)

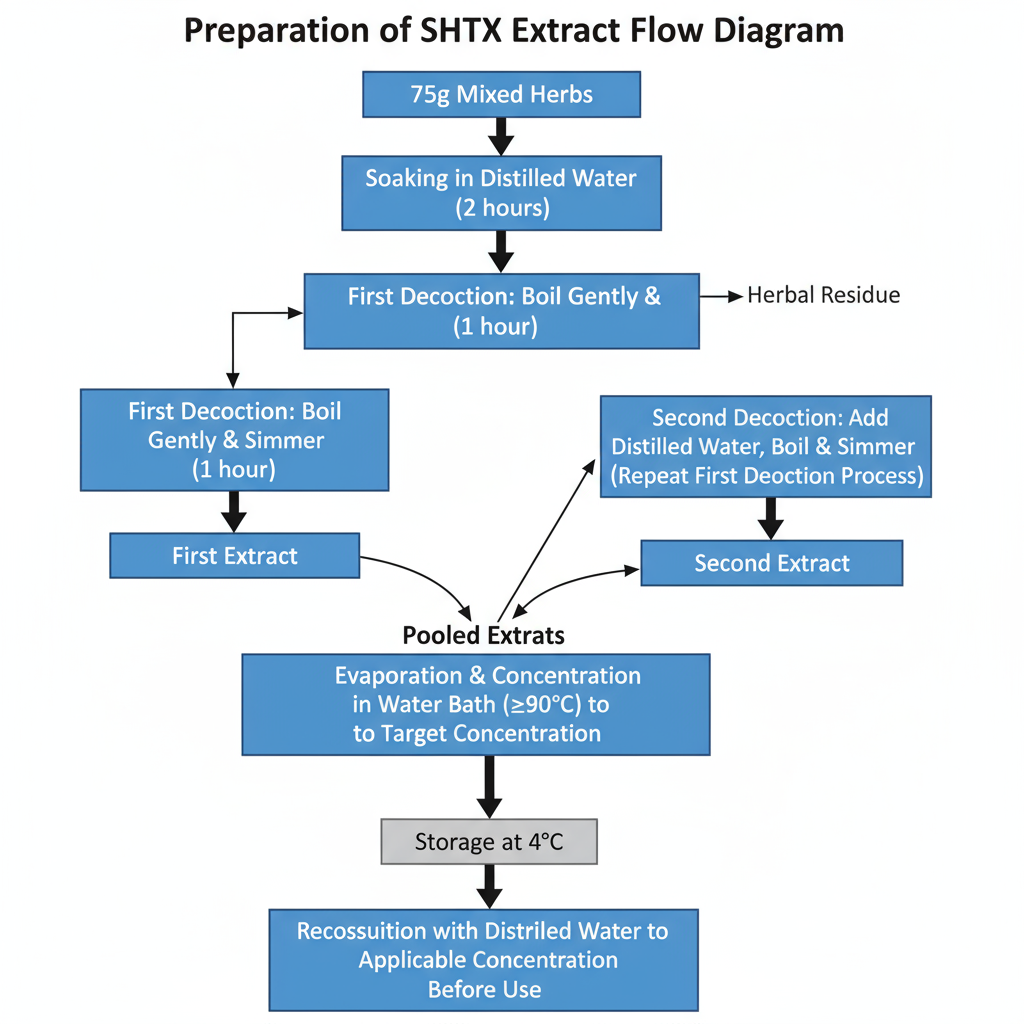

Supplement: Supplementary file 2 [file Image1.tif]
